# Temporary Kirschner wire fixation of the first metatarsophalangeal joint before osteotomy for hallux valgus

**DOI:** 10.1016/j.ijscr.2021.106104

**Published:** 2021-06-10

**Authors:** Ichiro Tonogai, Koichi Sairyo

**Affiliations:** Department of Orthopedics, Institute of Biomedical Science, Tokushima University Graduate School, 3-18-15 Kuramoto, Tokushima City, Tokushima 770-8503, Japan

**Keywords:** Hallux valgus, Temporary fixation, Kirschner wire, Metatarsophalangeal joint, Incongruency, Overcorrection

## Abstract

**Introduction:**

Numerous operative procedures have been described for correction of hallux valgus, including distal step-cut osteotomy such as the Mitchell osteotomy. However, overcorrection can occur due to technical problems with the initial metatarsal osteotomy. Here, we describe a modified Mitchell osteotomy with a novel method, the temporary Kirschner wire fixation of the first metatarsophalangeal joint (TeKFiM) method (Tonogai method), that can be used before osteotomy for hallux valgus to avoid incongruency and overcorrection.

**Operative technique:**

A skin incision and Y-shaped capsulotomy are performed and the medial exostosis is excised. Lateral capsule release is done if the first metatarsophalangeal (MTP) joint cannot be reduced manually. Next, a Kirschner wire (K-wire) is inserted subcutaneously through the medial side of the first proximal phalanx to the lateral side of the first metatarsal to preserve the correct congruency of the first MTP joint during surgery. To correct pronation of the distal fragment, step-off transverse cuts are made in the distal fragment, as described by Mitchell, reaching one-second to two-thirds of the transverse diameter of the neck from the plantar medial side. After the osteotomies are completed, the lateral spike of the proximal fragment is flattened. The distal fragment is displaced laterally and slightly plantarward, and the pronation deformity of the distal fragment is corrected by inserting a K-wire to act as a joystick. The osteotomy site is stabilized using two Herbert-type screws. After removal of the K-wire, the operation is completed by closing the medial capsule of the first MTP joint and the skin. A plantar cast is applied for 2 weeks, followed by a special heel brace for 4–6 weeks. Sutures are removed 2 weeks after surgery. Patients are allowed to start weightbearing gradually as tolerated from 2 weeks after surgery.

**Discussion:**

After osteotomy, it is difficult to maintain the correct congruency of the first MTP joint due to instability of the distal fragment. The TeKFiM method (Tonogai method) reliably maintains this congruency during surgery. Also, by using a K-wire as a joystick to fix the joint in correct congruency, the first toe is rotated and pronation is corrected by supinating the distal fragment. The K-wire also serves as a landmark for determining how far the distal fragment is shifted plantarward.

**Conclusions:**

We have developed a modified Mitchell osteotomy with the novel TeKFiM method (Tonogai method) before osteotomy for hallux valgus to avoid incongruency and overcorrection. This method also provides a landmark to correct pronation and plantarward shifting.

## Highlights

• Temporary Kirschner wire fixation of the first metatarsophalangeal joint (TeKFiM).
• TeKFiM is useful for hallux valgus surgery.
• TeKFiM method can avoid incongruency and overcorrection
• TeKFiM method make it easy to correct toe pronation.
• Kirschner wire serves as a landmark for plantarward shifting of distal fragment.

## 1 Introduction

Hallux valgus is a triplanar deformity of the first ray that is estimated to affect more than 20% of people aged 18–65 years and more than 35% of those aged ≥65 years [1]. More than 130 procedures have been described for the treatment of hallux valgus, including fusion of the first metatarsophalangeal (MTP) joint, excisional arthroplasty, distal first metatarsal osteotomy, and basal first metatarsal osteotomy. Mitchell's operation is a distal step-cut osteotomy through the neck of the first metatarsal that displaces the metatarsal head laterally to correct hallux valgus [2].

**Fig. 1:**
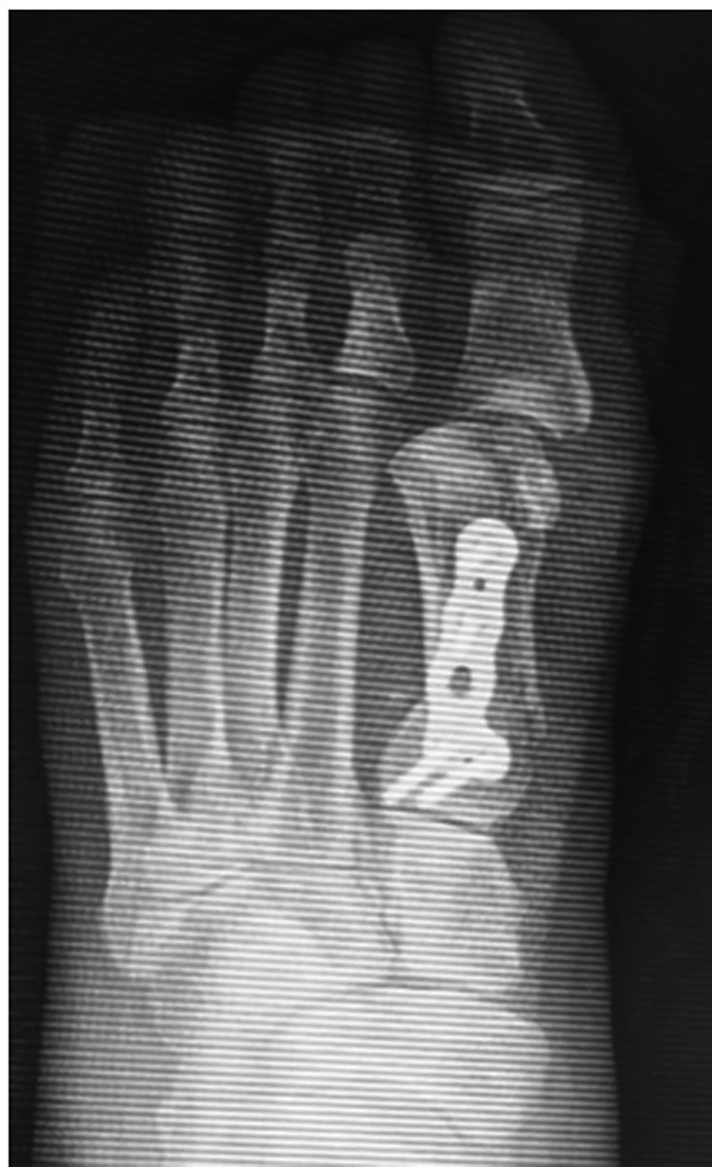
Radiograph showing incongruency of the first metatarsophalangeal joint due to overcorrection during surgery for hallux valgus, leading to hallux varus.

The reported frequency of iatrogenic hallux varus after hallux valgus surgery ranges from 2% to 13% [[3], [4], [5], [6], [7], [8]]. Suggested causes of iatrogenic hallux varus are related to overly aggressive attempts to correct hallux valgus. Excessive release of the lateral MTP joint capsule, excessive tightening of the medial MTP joint capsule, excessive resection of the medial eminence, and overcorrection of the intermetatarsal angle between the first and second metatarsal shafts (Fig. 1) are the most commonly reported causes [9]. However, no methods have been reported for preventing overcorrection during surgery for hallux valgus.

Here we describe a modified Mitchell osteotomy with a novel method, the temporary K-wire fixation of the first metatarsophalangeal joint (TeKFiM) method (Tonogai method), that can be used before osteotomy for hallux valgus to avoid incongruency and overcorrection. This has been reported in line with the PCROCESS 2020 criteria [10].

## 2 Operative technique

**Fig. 2:**
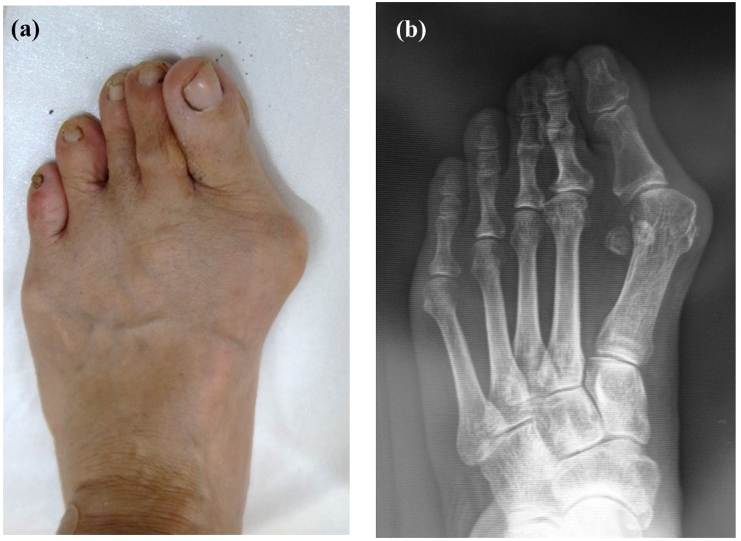
Preoperative photograph (a) and radiograph (b) of the foot in a patient with hallux valgus.

The patient whose images are used in this article to demonstrate the TeKFiM method (Tonogai method) gave informed consent for the publication of the images in this report. A preoperative photograph and radiograph are shown in Fig. 2a, b. The patient had no past medical history. The patient also had no family history of relevant genetic information and psychosocial history. The surgery was performed by I.T. who graduated from the medical university in 2004 and was a foot and ankle surgeon.

A short straight medial skin incision is made over the first MTP joint, extending along the first metatarsal distal shaft. After protection of the dorsal digital branch of the medial plantar nerve, a V-shaped incision with a distally based capsular flap is made in the medial joint capsule. The medial hyperostosis is excised. The tendon of the abductor hallucis muscle and a portion of its belly are retracted plantarward while the periosteum of the metatarsal shaft is incised and elevated.

**Fig. 3:**
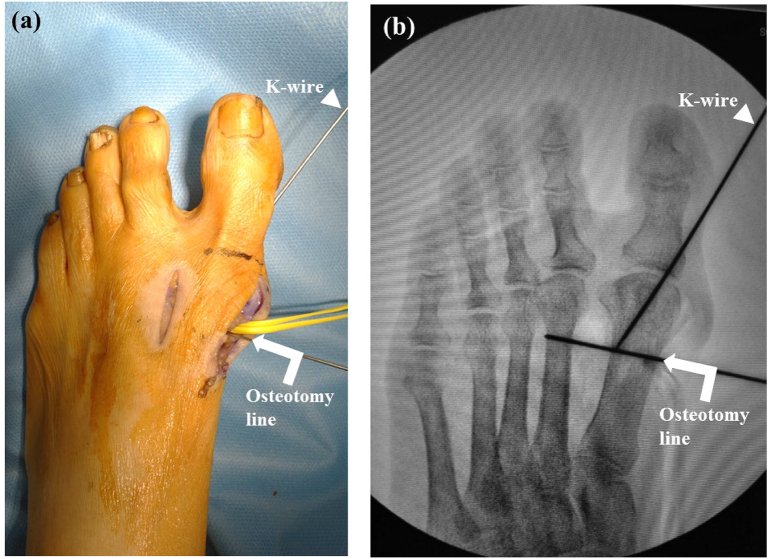
Photograph (a) and fluoroscopic image (b) showing insertion of the Kirschner wire (K-wire) (arrowheads) through the medial side of the first proximal phalanx to the lateral side of the first metatarsal subcutaneously to maintain correct congruency of the first metatarsal joint and avoid overcorrection during surgery after reduction of the first metatarsophalangeal joint. Arrows show the additional K-wire serving as a landmark for the osteotomy line.

Release of the lateral capsule by cutting the transverse metatarsal ligament and performing an adductor hallucis tenotomy is indicated if the subluxated first MTP joint cannot be reduced manually and an incongruent MTP joint is seen under fluoroscopy. After the first MTP reduction, a K-wire is inserted through the medial side of the first proximal phalanx to the lateral side of the first metatarsal subcutaneously to keep the correct congruency of the first MTP joint during surgery (Fig. 3a, b).

**Fig. 4:**
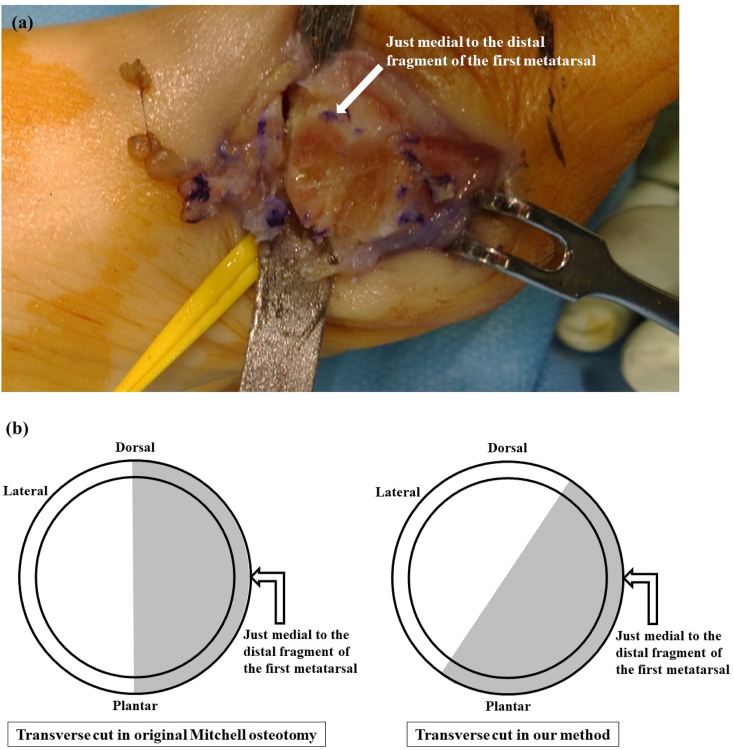
Photograph (a) and diagram (b) showing the distal cut made transverse to the first metatarsal and across approximately one-second to two-thirds of the width of the metatarsal shaft from the plantar medial direction, although not just medial in order to correct the pronation deformity. Arrows show just medial to the distal fragment of the first metatarsal. In the diagram, the proximal end of the distal fragment is seen from the proximal direction. Gray area shows the transverse area cut using either the original Mitchell osteotomy or our novel method.

**Fig. 5:**
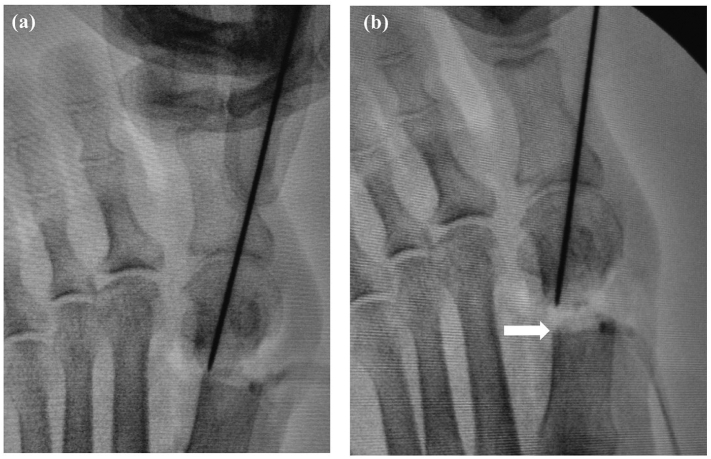
Fluoroscopic images obtained before (a) and after (b) flattening of the distal end of the proximal fragment of the first metatarsal. Arrow shows the flattened site.

**Fig. 6:**
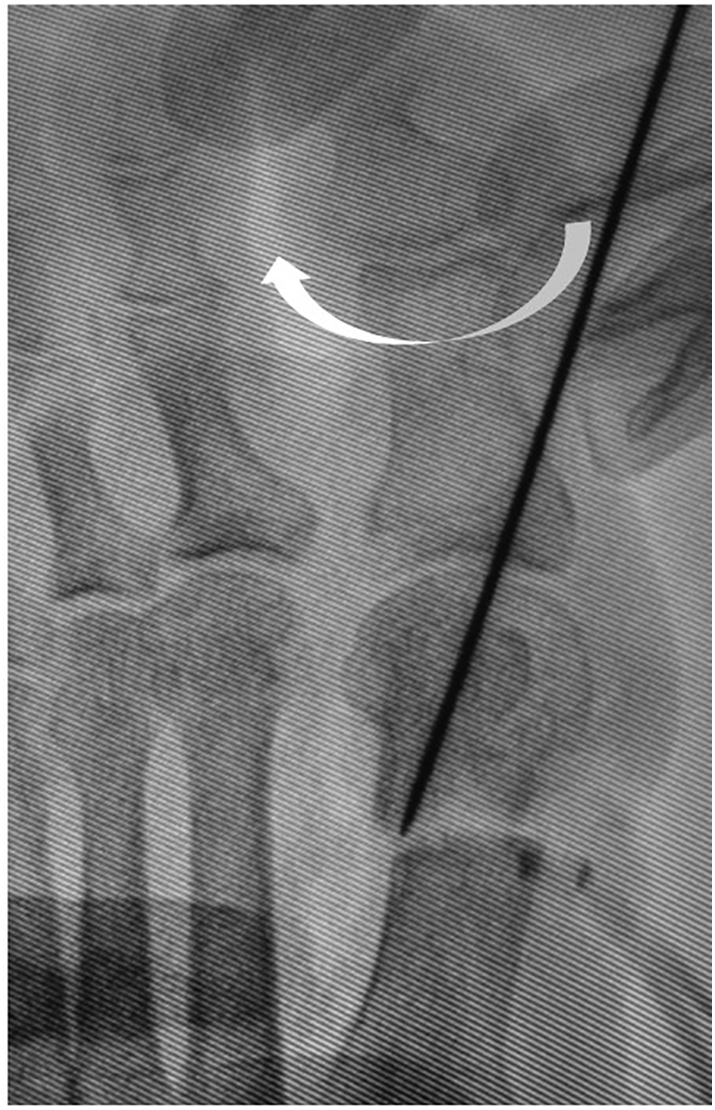
Fluoroscopic image showing rotation of the first toe and correction of pronation by supination of the distal fragment using a K-wire as a joystick to fix the first metatarsophalangeal joint in correct congruency.

Using Mitchell's osteotomy technique, parallel cuts are made at the metatarsal neck perpendicular to the long axis of the first metatarsal at 2-mm intervals using an oscillating saw. Starting medially, the distal cut is made transverse to the first metatarsal and across approximately one-second to two-thirds of the width of the metatarsal shaft from the plantar medial direction, although not just medial in order to correct the pronation of the distal fragment (Fig. 4a, b), which is different from the original Mitchell osteotomy. Second, the proximal cut is a complex osteotomy that begins 2 mm proximal to the distal cut on the medial aspect of the first metatarsal shaft. The inclination of the lateral spike of the distal end of the proximal fragment is flattened with a bone saw vertical to the second metatarsal (Fig. 5a, b), which is also different from the original Mitchell osteotomy. By using the K-wire as a joystick to fix the first MTP joint in correct congruency, the first toe is rotated and pronation is corrected by supinating the distal fragment (Fig. 6). The distal fragment is intentionally displaced laterally and slightly (1–2 mm) in the plantar direction to avoid transfer metatarsalgia. The inserted K-wire is also a useful landmark for determining how much the distal fragment is shifted in the plantar direction.

**Fig. 7:**
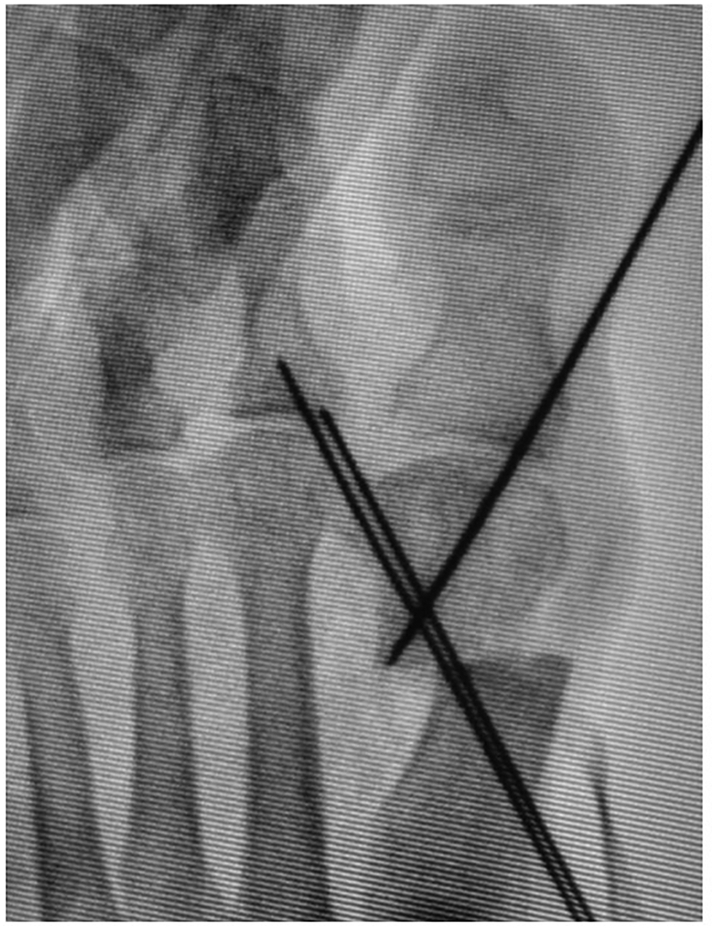
Fluoroscopic image showing insertion of the guide pin for the screw from the medial side of the proximal fragment in a lateral and distal direction.

**Fig. 8:**
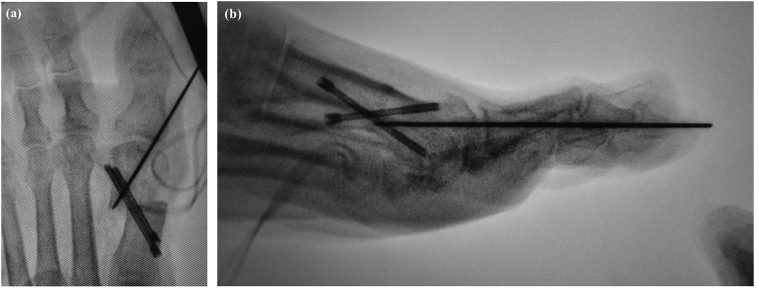
Fluoroscopic images in a frontal view (a) and lateral view (b) show placement of two Herbert-type screws of appropriate length across the osteotomy site to fix the distal fragment.

**Fig. 9:**
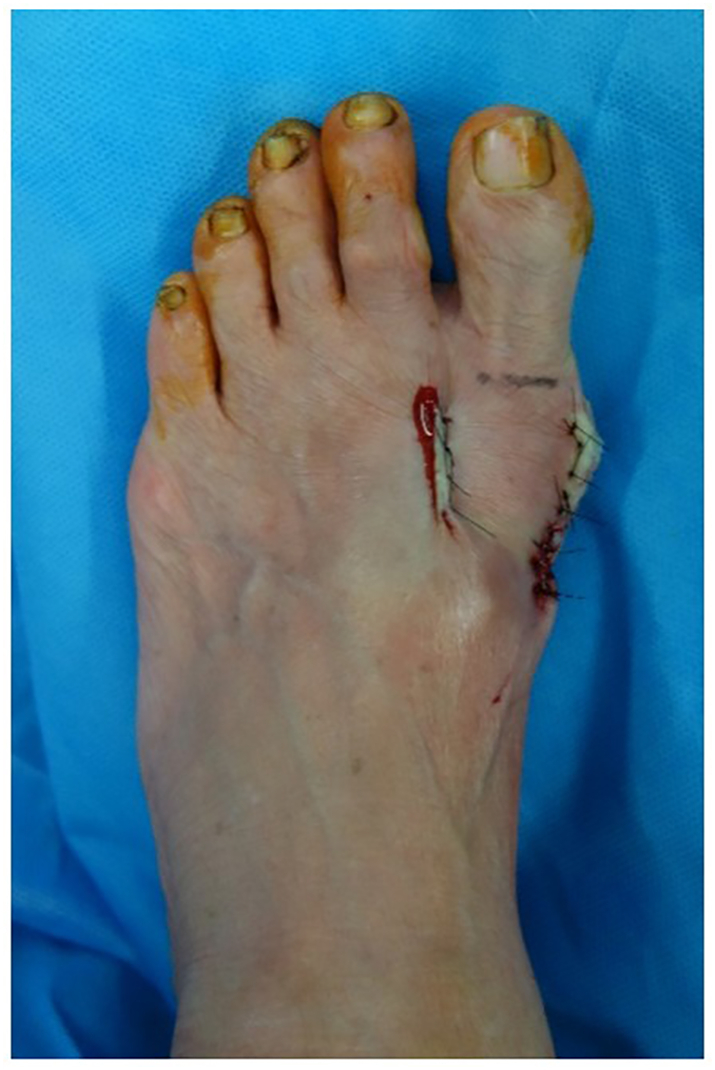
Photograph showing correct alignment of the first toe soon after surgery.

The guide pin for the screw is then inserted from the medial side of the proximal fragment and directed in a lateral and distal direction (Fig. 7). After checking the position under fluoroscopy to confirm that the inclination of the longitudinal axis of the first metatarsal is parallel to the longitudinal axis of the second metatarsal, we place two Herbert-type screws (2.5 or 3.0 mm diameter) of appropriate length across the osteotomy site, and the distal fragment is fixed (Fig. 8a, b). The tip of the screw approaches the opposite aspect of the head and its proximal end is approximately 1.5 cm proximal to the osteotomy site. The articular surface of the first metatarsal head is then inspected to ensure that the tip of the screw does not penetrate the articular surface. After removal of the K-wire used for temporary fixation of the first MTP, capsulorrhaphy of the joint is performed using absorbable sutures. The medial capsule is tightened to maintain straight alignment of the hallux with the first metatarsal bone, adjusting the correction with capsular tension (Fig. 9). An elastic bandage is applied between the first and second toes as a postoperative dressing to hold the first toe in the desired alignment. Active movement of the ankle is encouraged as soon as tolerated.

Postoperatively, a light compression bandage is applied for 2 days, and patients wear a plantar cast for 2 weeks after surgery, with no weightbearing, to allow sufficient time for soft tissue healing and adherence of the capsule to the raw bone surface at the bunionectomy site. Skin sutures are removed 2 weeks after surgery. Two weeks after the surgical splint is removed, patients are permitted to walk while wearing boots with specially designed heels and to gradually increase weightbearing. The boots are removed 6–8 weeks after surgery. Thereafter, patients can use normal walking shoes.

## 3 Discussion

This report describes the TeKFiM method (Tonogai method), which is a modification of the Mitchell osteotomy method used to correct hallux valgus. After osteotomy, it is difficult to maintain correct congruency of the first MTP joint because of instability of the distal fragment of the head of the first metatarsal. Using this novel method, the first MTP joint can be temporarily fixed before osteotomy to avoid incongruency and overcorrection during surgery.

Ferrari et al. performed a systematic review of the published literature and concluded that there was no compelling evidence of the superiority of any particular type of surgery over others to treat hallux valgus [11]. The Mitchell method is a distal step-cut osteotomy that is widely used to correct mild to moderate hallux valgus [2]. Various authors have demonstrated satisfactory correction in more than 80% of feet [2,[12], [13], [14]]. We have adapted the Mitchell procedure to include flattening of the distal end of the lateral spike of the proximal fragment of the first metatarsal vertical to the second metatarsal. This procedure might be effective for not only mild-to-moderate but also severe hallux valgus because it is useful for making the longitudinal axes of the first and second metatarsals parallel, and allows correction of moderate to severe deformity.

Pronation of the first toe is common in hallux valgus. Kim et al. reported that the average pronation of the first metatarsal was greater in patients with hallux valgus than in controls (21.9 degrees vs 13.8 degrees, respectively) [15]. We believe that this rotational pathology should be corrected because it seems to be a risk factor for postoperative recurrence. Therefore, we made a distal transverse cut in the distal fragment in the plantar medial side, although not just medial to the first metatarsal because it is necessary to correct pronation of the first toe. This step is not included in the original Mitchell osteotomy. By using a K-wire as a joystick to fix the first MTP joint in correct congruency, the first toe is rotated and pronation is corrected by supinating the distal fragment. Temporary fixation using a K-wire allows the plane of the first toenail to be compared with that of the other toe nails, which makes it easier to determine the ideal position of the first toe.

One of the disadvantages of Mitchell's method is shortening of the first ray, which causes dorsiflexion at the osteotomy site and leads to transfer metatarsalgia, which is associated with uncontrolled shortening of the first metatarsal by 6–7 mm in 7%–15% of cases [12,14,16,17]. Our TeKFiM method minimizes flattening of the lateral spike of the distal end of the proximal fragment and also shifts the distal fragment plantarward to prevent metatarsalgia. The K-wire also serves as a useful landmark for determining how far the distal fragment is shifted plantarward, comparing the first toe with the other toes.

Wu's modified Mitchell osteotomy involves firm fixation of the osteotomy site with two Herbert-type screws [18,19]. We agree with this recommendation because, besides excessive shortening of the metatarsal, metatarsalgia in dorsiflexion can also be caused by malunion of the distal fragment. Therefore, we also use Herbert-type screws to provide secure fixation at the osteotomy site. Moreover, there is no need for subsequent screw removal because the plate is sufficiently bulky. Intramedullary fixation seems to be rigid and we have had no breakages so far.

The TekFiM method has some limitations. Some patients with hallux valgus have a hypermobile first metatarsal-medial cuneiform joint, which is a contraindication to use of this method. An Akin-type procedure, such as osteotomy of the first proximal phalangeal joint, might be needed if the first toe shows severe pronation deformity. Clinical evaluation of the TeKFiM method in a sufficient number of cases over a longer follow-up period is necessary. A randomized study of this method should also be conducted in the future.

## 4 Conclusion

We have developed the TeKFiM method (Tonogai method) for use before osteotomy for hallux valgus to avoid incongruency and overcorrection. Our procedure is combined with a modified Mitchell osteotomy using two Herbert-type screws for fixation. This procedure is easy to perform and achieves good congruency. The K-wire inserted during this novel procedure serves as a useful landmark for correcting pronation and assessing how far the distal fragment is shifted plantarward.

## Declaration of competing interest

The Authors declare that there is no conflict of interest.
